# The diagnostic process from primary care to child and adolescent mental healthcare services: the incremental value of information conveyed through referral letters, screening questionnaires and structured multi-informant assessment

**DOI:** 10.1192/bjo.2022.47

**Published:** 2022-04-07

**Authors:** Semiha Aydin, Bart M. Siebelink, Matty R. Crone, Joost R. van Ginkel, Mattijs E. Numans, Robert R. J. M. Vermeiren, P. Michiel Westenberg

**Affiliations:** Department of Developmental and Educational Psychology, Leiden University, The Netherlands; Department of Child and Adolescent Psychiatry, Leiden University Medical Centre, The Netherlands; and Department of Public Health and Primary Care, Leiden University Medical Centre, The Netherlands; Department of Child and Adolescent Psychiatry, Leiden University Medical Centre, The Netherlands; Department of Public Health and Primary Care, Leiden University Medical Centre, The Netherlands; Methodology and Statistics Unit, Institute of Psychology, Leiden University, The Netherlands; Department of Public Health and Primary Care, Leiden University Medical Centre, The Netherlands; Department of Child and Adolescent Psychiatry, Leiden University Medical Centre, The Netherlands; and Youz, Parnassia Group, The Netherlands; Department of Developmental and Educational Psychology, Leiden University, The Netherlands

**Keywords:** Evidence-based assessment, primary care, psychological testing, diagnostic decision making, secondary mental healthcare

## Abstract

**Background:**

A variety of information sources are used in the best-evidence diagnostic procedure in child and adolescent mental healthcare, including evaluation by referrers and structured assessment questionnaires for parents. However, the incremental value of these information sources is still poorly examined.

**Aims:**

To quantify the added and unique predictive value of referral letters, screening, multi-informant assessment and clinicians’ remote evaluations in predicting mental health disorders.

**Method:**

Routine medical record data on 1259 referred children and adolescents were retrospectively extracted. Their referral letters, responses to the Strengths and Difficulties Questionnaire (SDQ), results on closed-ended questions from the Development and Well-Being Assessment (DAWBA) and its clinician-rated version were linked to classifications made after face-to-face intake in psychiatry. Following multiple imputations of missing data, logistic regression analyses were performed with the above four nodes of assessment as predictors and the five childhood disorders common in mental healthcare (anxiety, depression, autism spectrum disorders, attention-deficit hyperactivity disorder, behavioural disorders) as outcomes. Likelihood ratio tests and diagnostic odds ratios were computed.

**Results:**

Each assessment tool significantly predicted the classified outcome. Successive addition of the assessment instruments improved the prediction models, with the exception of behavioural disorder prediction by the clinician-rated DAWBA. With the exception of the SDQ for depressive and behavioural disorders, all instruments showed unique predictive value.

**Conclusions:**

Structured acquisition and integrated use of diverse sources of information supports evidence-based diagnosis in clinical practice. The clinical value of structured assessment at the primary–secondary care interface should now be quantified in prospective studies.

The formulation of a clinical diagnosis is critical to child and adolescent mental healthcare (CAMH).^1–3^ The current approaches for the diagnostic process include the judgement of a clinician or the use of structured assessment instruments. Four decades of research support the use of structured instruments, which results in more consistent application of diagnostic criteria, a decrease in information variance and bias, and improved recognition of less obvious or secondary conditions.^4–6^ Clinical and evidence-based assessment (EBA) guidelines therefore recommend integration of both methods, to benefit from the nuance and parsimony associated with clinical judgement, combined with the accuracy and reliability intrinsic to structured assessment.^7,8^ As in clinical practice with stepped-care and matched-care approaches, assessment is conducted in sequential stages; with EBA the question is raised as to whether instruments meaningfully contribute to the diagnostic work-up and how far each additional information step overlaps. Although the various instruments have been studied for value as standalone measures,^7^ less is known about the incremental value of the various nodes of information. Given the tension between efficiency of information gathering and reliability in the diagnostic process,^9^ a better understanding is needed of the value of a validated diagnostic work-up; in this case, a work-up that captures the combined benefits of structured assessment and clinical judgement, suggesting potential for use at the interface between primary and secondary CAMH. Accordingly, the aim of the present study was to investigate the incremental value of routinely gathered successive assessments. We investigated the added value of referral letters, a screening questionnaire and a structured multi-informant assessment gathered during the registration procedure at an academic centre for child and adolescent psychiatry.

## The diagnostic procedure

In several countries, it is standard practice for CAMH registration to take place via front-line practitioners such as paediatricians or general practitioners. If a decision is made, based on screening or clinical judgement, to refer to CAMH, a referral letter indicating the probable mental health diagnosis forms a bridge to CAMH. For many children and adolescents, referral letters represent the only form of information transfer from the referrer, and may contribute to the diagnostic and treatment process in CAMH. Although many professionals in the field believe that referral letters have no clinical value, in a recent study, we found that 42–93% of youth reasons for referral saw no change in later psychiatric diagnosis.^13^ Although these numbers are substantial, we also observed considerable variation between disorder groups, with internalising problems in particular showing a relatively poor detection accuracy.

In EBA, a decision to refer should follow administration of a screening instrument. This procedure allows for the common false positives of screening instruments to be corrected by clinician judgement, and acknowledges that screening often helps improve detection of less obvious problems such as internalising disorders, thereby improving adequate referrals and access to treatment. Regrettably, the use of screening instruments is infrequent, a problem often attributed to the limited time available for patient consultation.^14^ Many of the current short screening questionnaires were specifically developed to address this problem. Unintendedly, development of these questionnaires may have further limited their implementation, because understanding the pros and cons of the wide array of current screening instruments, together with interpretation of outcomes, has become more challenging.^14–17^ A recent review of accessible instruments identified 672 questionnaires, of which only four broad screening instruments qualified as brief, short, free, and with excellent psychometric characteristics.^17^ One of these instruments is the Strengths and Difficulties Questionnaire (SDQ), available in over 70 languages.^18^ The SDQ was found to be as reliable and feasible as the much lengthier Achenbach scales (the Youth self report (YSR), Child behavior checklist (CBCL) and Teacher report form (TRF)) that are frequently used in many European countries.^19–21^ The developers of the SDQ proposed using the instrument before a clinical appointment, as a guide to decision-making.^22^ However, regarding recognition of emotional problems, studies suggest that the SDQ might be insufficient, a problem likely related to the limited number of questions in the scale, differences in study samples and general difficulties in detecting internalising disorders.^18,23^

The detection of mental health problems, including internalising problems, often improves with the use of more extensive assessment methods. In EBA, more extensive assessment methods are in fact recommended in the case of individuals with high scores during screening. The Development and Well-Being Assessment (DAWBA) instrument combines the responses of various informants (adolescents, parents and/or caregivers and teachers) to closed-ended questions into so-called DAWBA band scores that indicate the likelihood of a child having any of 17 common mental health disorders.^22,24^ The DAWBA band scores were envisioned as a way to avoid the costly involvement of a clinician, and to be a pragmatic solution for common issues at the point of care. Nonetheless, the value of DAWBA bands when accounted for the value of screening and clinical judgement in primary care is not yet investigated.

As part of the DAWBA, informants are also prompted to describe their problems and the context of their problems in their own words. These are then evaluated by a clinician, who integrates the various factors to form a relatively nuanced image without the high cost of a full interview with a specialist clinician. DAWBA clinician ratings were found to be conservative regarding the number of diagnoses made when compared with elaborate diagnostic interviews.^25^ Studies of the clinician-rated DAWBA found that it was useful in reducing unnecessary referral for externalising disorders, and that it highlighted internalising disorders that would not have been detected otherwise.^26,27^ Nevertheless, the exact extent to which clinician ratings supplement information from a primary care clinician, screening results and automatised DAWBA probability band scores remains an important but unanswered question.

## Aims

In summary, the feasibility and psychometric properties of the DAWBA and SDQ have been individually well-researched in community, clinical and research settings in various European countries. However, less information is available regarding the predictive value of instruments when taking into account the usual overlap of information gained during successive steps in EBA. The aim of the present study was to determine both the unique and incremental predictive values for four sources of information in predicting a medical record consensus diagnosis: referral letters, a screening questionnaire (SDQ^18^), a more elaborate structured assessment (DAWBA band scores^22^) and the remote evaluation of structured and unstructured responses by a clinician (the clinician-rated DAWBA). We hypothesised that each instrument would show incremental value in predicting the classification of five disorder groups commonly treated in CAMH: anxiety, depression, autism spectrum disorders (ASD), attention-deficit hyperactivity disorder (ADHD) and behavioural disorders.

## Method

### Data source and procedure

The starting point for the sample was children and adolescents who were referred to Leiden University Medical Centre Curium (LUMC Curium). LUMC Curium is an in-patient and out-patient mental health clinic delivering specialised care to young people aged 3–18 years.

About 70% of the yearly case-load at the institution consists of out-patient referrals that follow a routine procedure, including referral letters, the SDQ and DAWBA. The remainder consists of in-patient referrals that follow a referral intake procedure adapted to cases in need of urgent evaluation, in which case questionnaires are not completed at registration. We included young people who registered between January 2015 and December 2017; followed the routine procedure, including the SDQ and DAWBA; and had an accessible referral letter in the medical record. The procedures used to extract and code referral letters are described in detail in our recent publication on referral letter general practice.^13^ To briefly summarise, using an iterative process, we created a manual to extract and code text in referral letters. The manual was then tested for interrater reliability by authors S.A., M.R.C., B.M.S. and P.M.W. (κ = 0.77–0.90). We did not differentiate symptoms indicated in referral letters from suggested diagnoses. For instance, when an referral letter reported ‘treatment for anxiety disorders?’ or ‘fearful’, both were coded as an indicator of the category anxiety disorders and related problems. Multiple indications were often found in referral letters and were thus coded. However, <20% of referral letters indicated more than four problems,^13^ which was also the case in the current sample.

The LUMC Medical Ethical committee waived a need for informed consent because of the retrospective nature of the study (approval number G18.080). Furthermore, the data management plan was approved by the scientific committee of the LUMC Departments of Public Health and Primary Care, LUMC Curium Department of Child and Adolescent Psychiatry and the Institute of Psychology at Leiden University.

### Measures

All measures were extracted from medical records. We extracted referral letters as they were scanned and filed in individual patient medical records. The SDQ, structured DAWBA data and classifications that are also outcome measure were extracted simultaneously from the medical record system.^28^

In The Netherlands, only a healthcare professional can make a formal referral to youth and adolescent psychiatry, which then proceeds via either general practice, specialised healthcare (hospitals) or youth welfare offices (also called local youth teams). We did not include the type of professional as a covariate in the main analyses, as initial logistic regression analyses showed wide confidence intervals and no statistically significant differences between the various types of referrers.

#### Structured assessment: SDQ and DAWBA

During registration, families are provided with unique login codes for the online DAWBA package, which can be completed by up to two parents or caregivers, the young person themselves (if aged >11 years) and up to two teachers. The package always starts with the SDQ, and then moves on to the DAWBA instrument. Rules regarding skipping come into play when an informant shows low scores on a conceptually related SDQ scale and provides negative answers to a gate-keeping question at the beginning of each DAWBA chapter.^22^ In the DAWBA package, SDQ scale scores and DAWBA probability band scores are generated for each informant individually, and subsequently integrated into an overall SDQ score for each scale (0, 1, 2) and a DAWBA probability band score for each chapter (0–5). The cut-off scores and rules concerning integration of informant's scores can be found at www.sdqinfo.org and www.dawba.net. If not otherwise specified, we used integrated scores for all analyses. To analyse whether each assessment method indicated the presence of a disorder group, we dichotomised scores by separating the upper two scores from the lower score(s).^24,27,29^

##### SDQ

The SDQ covers four problem areas (emotional, conduct, hyperactivity and peer problems scales) across 20 items, asks about children's strengths in five items (prosocial scale), and the impact and burden of problems in eight items. Informants rate items on a three-point Likert scale (0 = not true, 1 = somewhat true, 2 = certainly true), with higher scores indicating more problems. Although the SDQ was not formally created to give indications of a probable ASD, in a later study, Goodman et al^30^ proposed use of a difference score by subtracting the total for the peer problems scale from the score for the prosocial scale. We calculated this difference score solely based on parental scores, as the few studies available suggest that parents show the highest accuracy in detecting ASD.^23,31,32^

##### DAWBA probability band scores

The DAWBA^22^ estimates the likelihood of the presence of 17 common mental health disorders. These so-called probability bands are automatically generated in the online DAWBA environment by integrating various informant responses to closed-ended questions.^24^ The questions are linked to the DSM criteria and result in probability band scores of 0, 1, 2, 3, 4 and 5, corresponding to prevalences found in the original British epidemiologic sample and approximating likelihoods of <0.1%, 0.5%, 3%, 15%, 50% and >70%.^24^ Thus, a probability band score of 5 suggests that 70% or more of the cases with a similar response profile to the British reference sample were found to have that diagnostic outcome. When the DAWBA did not produce a score for a disorder group (e.g. behavioural disorders), we took the highest probability band score among the more specific disorders (i.e. the highest score among conduct and oppositional deviant disorder).^24^

##### Clinician-rated DAWBA

Informants are also prompted to describe problems and their context in their own words. A senior clinical psychologist evaluated the open-ended questions, together with the SDQ and DAWBA probability band results, and scored the likelihood of a disorder on a three-point scale (absent, unsure, present). This final stage facilitates the incorporation of the diverse strands of information to develop a nuanced image without the accompanying cost of visiting a specialist clinician. The next step is to add a short report to a patient medical record, to guide prioritisation of appointments and prevent tunnel vision during a face-to-face intake. In some study reports, clinician ratings are referred to as a DAWBA research diagnosis. In this paper, however, we use the term clinician-rated to prevent confusion with the outcome classification.

#### Clinical classification

The primary outcome measure was a patient's digital medical record classification according to the Longitudinal, Expert, All Data (LEAD) procedure.^12^ This is a product of all collected information and clinical judgement, including patient and family history, mental health treatment history, structured assessment and, if necessary, process diagnostics and additional assessment methods depending on suspected differential diagnoses.^10,33^ Based on these insights, a case conceptualisation is formed as a basis for treatment initiation, and a classification selected and entered into the patient's medical record. Up to five different classifications could be recorded per case, and all were extracted for this study.

#### Missing data

SDQ scale scores were available for all cases and DAWBA band scores were available for 97.7–98.9% of cases (depending on disorder group), but clinician-rated DAWBA data were available for only 52.1% of cases, as DAWBAs were not evaluated by a clinician during the first half of the study period. As this was a result of management decisions and unrelated to our research question, we could assume the data to be missing at random. To reliably estimate missing data, we applied multiple imputation (with *m* = 100) using the *mice* package in the R environment.^34–38^ Multiple imputation creates multiple sets with plausible values for missing cells, by drawing values from the observed cases and predicting from other associated variables in a data-set. Hence, it minimises bias relative to complete-case analysis. Generating multiple data-sets enables estimation of the uncertainty in the imputation process compared with, for example, simple mean imputation. In multiple imputation, it is necessary to balance the number of predictors and observed cases, as with regression analyses in general. Therefore, we limited the number of predictors during multiple imputation, such that a minimum number of 15 cases had to be observed for each contributing predictor.

### Statistical analysis

In the statistical analysis, we first computed diagnostic metrics such as sensitivity and specificity for each instrument. Next, we inspected youth diagnostic trajectories through the current sequence of four methods. To this end, we cross-tabulated frequencies of positive and negative indications in a four-layer table, with each of the methods and the diagnostic outcome. To examine the effect of each added predictor on model fit, likelihood ratio tests^39^ were performed with the *D3()* function in *mice*.^34^ Multiple logistic regression analyses were performed, with each of the five diagnostic groups as the outcome and the assessment methods as the predictor, to quantify unique and corrected predictive values. Diagnostic odds ratios of the instruments were extracted from the univariable and multivariable logistic regression models.

## Results

The sample age ranged between 5 and 18 years (mean 11.08, s.d. 3.45) and 57.4% were boys (Table 1).

**Table 1:** Sample characteristics (*N* = 1259)

	*n* (%)
Age, years	
5–9	474 (37.6)
10–14	508 (40.4)
15–18	277 (22.0)
Gender	
Male	723 (57.4)
Female	536 (42.6)
CGAS	
20–40	83 (6.6)
41–50	503 (40.0)
51–60	514 (40.8)
>61	96 (7.6)
Missing	63 (5.0)
Medical conditions	
None classified	958 (76.1)
Singular	99 (7.9)
Complex	52 (4.1)
Missing	150 (11.9)
Number of clinical classifications (comorbidity)	
0	175 (13.9)
1	544 (43.2)
2	368 (29.2)
3	125 (9.9)
4	35 (2.8)
5–6	12 (1.0)
Type of clinical classifications	
Neurodevelopmental disorders	727 (57.7)
Schizophrenia spectrum and other psychotic disorders	2 (0.2)
Depressive disorders	134 (10.6)
Anxiety disorders	174 (13.8)
Obsessive–compulsive and related disorders	13 (1.0)
Trauma and stressor-related disorders	68 (5.4)
Somatic symptom and related disorders	42 (3.3)
Feeding and eating disorders	54 (4.3)
Elimination disorders	19 (1.5)
Gender dysphoria	7 (0.6)
Disruptive, impulse-control and conduct disorders	71 (5.6)
Substance-related and addictive disorders	2 (0.2)
Personality disorders	49 (3.9)

Distributions of the clinical classifications in the sample are depicted based on the higher-order chapters of the DSM-5 (e.g. ‘Neurodevelopmental disorders’). The number of clinical classifications is depicted on the level of the specific classifications (e.g. attention-deficit hyperactivity disorder and autism spectrum disorders). CGAS, Children's Global Assessment Scale score.

### Univariable diagnostic metrics

The diagnostic metrics of the assessment methods as standalone measures are depicted in Table 2. The sensitivity and specificity of the successive assessment tools varied per mental health disorder. The value of referral letters in detecting patients with anxiety disorders was relatively low compared with the other disorder groups and other instruments: 46.9% of those eventually classified with an anxiety disorder had been indicated as such in referral letters. However, referral letters showed a relatively high specificity in excluding minors without the condition (85.9%). The highest sensitivity regarding anxiety disorders was found for the SDQ (95.1%), but was accompanied by a risk of being overinclusive (specificity 22.9%; false discovery rate 85.2%, Supplementary material available at https://doi.org/10.1192/bjo.2022.47). The SDQ and referral letters showed the highest sensitivity and specificity, respectively, whereas the DAWBA probability band and the clinician-rated DAWBA showed a more balanced profile.

**Table 2:** Two-by-two cross-tabulation of the instruments per disorder group

		Anxiety disorders		Depressive disorders		ASD		ADHD		Behavioural disorders	
		+	−	+	−	+	−	+	−	+	−
Referral letters	**+**	38 (46.9)	81 (14.1)	39 (60.0)	73 (12.4)	108 (54.8)	89 (45.2)	114 (55.9)	99 (22.0)	26 (59.1)	156 (25.5)
	−	43 (53.1)	492 (85.9)	26 (40.0)	516 (87.6)	89 (45.2)	361 (80.2)	90 (44.1)	350 (78.0)	18 (40.9)	455 (74.5)
SDQ	**+**	77 (95.1)	442 (77.1)	62 (95.4)	457 (77.6)	140 (71.1)	204 (45.3)	181 (88.7)	230 (51.2)	38 (86.4)	328 (53.7)
	−	4 (4.9)	131 (22.9)	3 (4.6)	132 (22.4)	57 (28.9)	246 (54.7)	23(11.3)	219 (48.8)	6 (13.6)	283 (46.3)
DAWBA band	**+**	57 (70.4)	185 (32.2)	45 (69.2)	94 (16.0)	18 (9.1)	5 (1.1)	121 (59.3)	78 (17.4)	16 (36.4)	225 (36.9)
	−	24 (29.6)	388 (67.7)	20 (30.8)	495 (84.0)	179 (90.9)	445 (98.9)	83 (40.7)	371 (82.6)	28 (63.6)	384 (63.1)
Clinician-rated DAWBA	**+**	62 (76.5)	194 (33.9)	49 (75.4)	104 (17.7)	151 (76.6)	154 (34.2)	170 (83.3)	158 (35.2)	26 (59.1)	200 (32.7)
	−	19 (23.5)	379 (66.1)	16 (24.6)	485 (82.3)	46 (23.4)	296 (65.8)	34 (16.7)	291 (64.8)	18 (40.9)	411 (67.3)

Frequency (%) of the positive and negative indications made per instrument and per disorder group, as a ratio of the total number of positive and negative cases. Number of diagnoses and sample size were as follows: anxiety disorders *n* = 81 and *N* = 654; depressive disorder *n* = 65 and *N* = 654, ASD *n* = 197 and *N* = 647; ADHD *n* = 204 and *N* = 653; behavioural disorders *n* = 44 and *N* = 655. ASD, autism spectrum disorders; ADHD, attention-deficit hyperactivity disorder; SDQ, Strengths and Difficulties Questionnaire; DAWBA band, Development and Well-Being Assessment probability band score.

We found that all instruments except the SDQ performed similarly in discriminating minors with or without depressive disorders (Table 2). In line with earlier studies, the SDQ frequently gave a positive indication in this clinical sample, yet often for the wrong persons (specificity 22.4%).

Upon inspecting the metrics for ASD, the low number of positive indications by the DAWBA probability band was remarkable. Although the bands indicated ASD infrequently, they did so for genuine cases, resulting in a high positive predictive value (78.3%, Supplementary material) but low sensitivity (9.0%). The SDQ difference score (peer problems – prosocial score, see Methods) showed the highest sensitivity for ASD compared with other instruments. In contrast to high false positives for anxiety and depressive disorders, the SDQ showed a better specificity for ASD (54.7%). Referral letters and clinician-rated DAWBA scores showed a fairly even balance of sensitivity and specificity for ASD.

When considering ADHD, most instruments showed values similar to those for ASD, with the DAWBA probability band showing the best performance in the detection of ADHD (sensitivity 59.3%).

Behavioural disorders were frequently indicated by all instruments, yet seldom classified. This resulted in a very low predictive value. This frequent indication of behaviour problems resulted in relatively high sensitivity (86.4%).

After inspecting single descriptives, we explored frequencies of the instrument's successive positive and negative indications to gain insight into the potential of the sequence for prognostic use. Of the youth with an anxiety disorder indicated by all four instruments, 48.8% were eventually classified with anxiety disorders (Supplementary material). The classification rate was 54.9% for four successive indications of depressive disorders, 85.7% for ASD, 70.0% for ADHDs and 10.7% for behavioural disorders.

When we considered the predictive value of successive negative indications, we found that 98.2% of those negative on all four instruments were not classified to anxiety disorders, 98.3% were not classified to depressive disorders, 90.5% were not classified to ASD, 95.8% were not classified to ADHD and 99.1% were not classified to behavioural disorders.

### Incremental and independent predictive values

When we examined the incremental value of the four assessment tools relative to each other, successive addition of a following instrument resulted in improvement in model fit for nearly all of the (4×5) models (Table 3). Only the fit for behavioural disorders did not improve with addition of the clinician-rated DAWBA scores to the model (*P* = 0.82).

**Table 3:** Likelihood ratio test values comparing the effect of addition of instruments on model fit per disorder group

	Referral letters	+SDQ	+DAWBA band	+Clinician-rated DAWBA
Anxiety disorders	92.74	33.47	41.81	15.1
Depressive disorders	136.81	8.28*	39.63	17.48
ASD	166.29	44.48	15.25	14.50
ADHD	203.53	79.52	42.23	39.58
Behavioural disorders	44.26	16.04	16.78	0.02**

Likelihood ratio test results depicting change in model fit by successive addition of the instruments, computed in the imputed data-set. All values are significant at the *P* < 0.001 level, except *P = 0.004 and **P = 0.82. Note the low frequency of four successive positive indications for ASD and ADHD, as it was uncommon for these minors to have positive scores on all four instruments. SDQ, Strengths and Difficulties Questionnaire; DAWBA band, Development and Well-Being Assessment probability band score; ASD, autism spectrum disorders; ADHD, attention-deficit hyperactivity disorder.

**Fig. 1:**
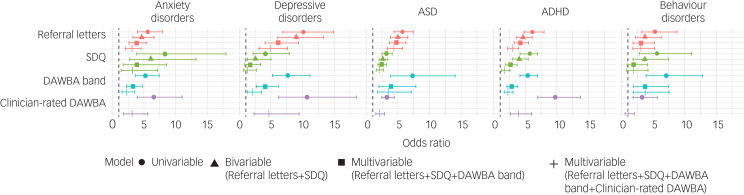
Univariable and multivariable odds ratios per instrument and per diagnostic outcome. Odds ratios per instrument and per disorder group for four models, computed in the imputed data-set. Each successive model contains one more instrument as a predictor, presenting how the odds ratios change when controlling for overlap with more instruments. The vertical line presents an odds ratio equal to 1. DAWBA band refers to the DAWBA probability band score. ADHD, attention-deficit hyperactivity disorder; ASD, autism spectrum disorder; DAWBA, Development and Well-Being Assessment; SDQ, Strengths and Difficulties Questionnaire.

By controlling for the value of up to three other instruments, we explored independent associations of the four instruments with the outcome classifications (Fig. 1). In these multivariable models, most instruments showed predictive value. Only in the case of the SDQ did we see a failure to improve the prediction of depressive disorders and behavioural disorders (depressive disorders: odds ratio 1.24, 95% CI 0.58–2.62; behavioural disorders: odds ratio 1.85, 95% CI 0.82–4.16).

For most disorder groups and instruments, we found no differences in magnitude of the associations in the multivariable models compared with the univariable prediction models. Similarly, no difference in patterns was observed when inspecting differences in the predictive value of the earlier instruments compared with the later instruments. The clinician-rated DAWBA, for instance, did not show consistently higher predictive values compared with the referral letters.

## Discussion

To the best of our knowledge, this study is the first to compare the predictive value of referral letters, broad band screening, structured multi-informant assessment and a clinician's remote evaluation in predicting diagnostic outcome in a single population. We found that all four nodes of assessment generally showed a positive contribution to the prediction of common child and adolescent mental health problems. Referral letters and SDQ scale scores showed either a high sensitivity or a high specificity, whereas DAWBA probability bands and clinician ratings were more balanced in terms of sensitivity and specificity. Referral letters performed especially well for depressive disorders, which might be related to an earlier observation made during the pilot phase of our previous study: professionals might focus on mood problems and associate it with risk of suicidal ideation.^40^ For the other disorder groups, referral letters showed better performance in terms of specificity compared with sensitivity. The SDQ, by contrast, was overinclusive, particularly for emotional problems;^23^ a finding in line with earlier conclusions that advised against complete reliance on the SDQ to guide referrals.^21^ To determine whether this might be a result of our categorisation of the SDQ indication as positive from the upper two scores, we reanalysed the data categorising only the upper category as positive. This resulted in a sensitivity decrease of 15 percentage points (to 80.5 for anxiety and 78.5 for depressive disorders), whereas specificity doubled to around 50% false positives. Nonetheless, compared with the other instruments, SDQ screening was still overinclusive, an issue inherent to a screening instrument's function (to detect problems), the clinical population, and, as underlined in the introduction, screening instruments should be accompanied by clinical judgement.

Although the SDQ does not officially have an ASD scale, we also included children and adolescents with ASD in the study to shed light on the issue of EBA in this clinically widespread population. We used a difference score suggested by the SDQ developers^30^ and found that children and adolescents with ASD were detected at a similar rate to other problem types on conceptually related SDQ scales. However, other studies have used other computational methods,^23,32,41,42^ and the respective methods have not yet been compared.

We also inspected frequencies of successive positive and negative indications as a first approach to the question of outcomes for young people who show positive or negative scores on a sequence of assessment instruments. In this explorative inspection, we found that four successive indications of anxiety or depressive disorders resulted in only a one in two chance of being classified to these outcomes. By contrast, when all instruments indicated ASD or ADHD, cases were indeed clinically classified as such. Regarding behaviour problems, we found that even four successive positive indications were not predictive of a classification to behavioural disorders. When considering the opposite situation, those with four successive negative indications, we found that about 1% was classified to anxiety, depressive or behavioural disorders, whereas around 5% or 10% were still classified to ADHD or ASD, respectively. It is unsurprising that rates were highest for ASD, because if initial instruments fail to suggest this relatively difficult diagnosis further clinician based investigations subsequently detect ASD. These results underline the need for elaborate diagnostics, the inclusion of clinicians when aiming for specialised treatment and the importance of future studies with a diverse sample for better generalisability.

We found added benefits with each successive node of assessment, with only one exception for one outcome: the clinician ratings showed no improvement in the prediction of behavioural disorders relative to the three previous instruments combined. This might be because of the already marginal prediction of behavioural disorders and the relatively conservative properties of the clinician-rated DAWBA.^25^ With regards to the independent predictive value, we found that nearly all instruments remained individually associated with the outcome even when corrected for overlap with other instruments. Only the SDQ showed no independent value in predicting depressive and behavioural disorders when corrected for information provided by other nodes of assessment. In contrast to general literature suggesting that instruments applied later in a sequence might show stronger effects,^43^ we observed no increase in effect. Therefore, the study results give no support for use of the most elaborate instrument first and only, and support a stepwise approach to assessment.^44^

### Limitations

Although this study presented unique data on an important question, some limitations should be kept in mind. First, people involved in classifying outcomes were not blinded to the instrument's results. To what extent results were viewed when formulating a diagnosis is not known. As regards the effect of the availability of DAWBA data, for instance, there are indications that it improves decision-making in the case of internalising problems, but not in the case of externalising problems.^27^ In an effort to explore this type of potential effect, we split the sample between those with or without clinician ratings (see Methods), but did not find differences in odds ratios between subsamples. Regardless, if disclosure had any effect it would likely result in the presented odds ratios overestimating associations. Looked at more positively, our research question concerned the relative predictive value of the instruments and, in principle, all instruments were accessible and have shown predictive value, also in other studies with blinding.

Another limitation concerns discriminant ability of the instruments. If the aim is to predict the type and classification of a problem, insight into how scales relate to conceptually parallel classifications is not sufficient. Future studies could therefore focus on the discriminant ability of the tools and investigate cross relations between scales and types of problems. Furthermore, we focused only on the type of problems, whereas taking the staging and impact of symptoms into account could benefit clinical practice.^45^

### Implications

The questions addressed in this study are directly relevant to clinical practice. Referral letters are, by definition, available for many cases, yet are seldom incorporated into the diagnostic process. In this study, we found that referral letters add value, even when corrected for overlap with structured assessment instruments. Similarly, the DAWBA package has the potential to ease the assessment process by capturing the SDQ as a short yet sensitive screening instrument, the DAWBA structured questions as a broad assessment tool to ‘cast a wide net regarding the presenting problem of a client’,^11^ and the clinician-rated DAWBA to add some nuance regarding the fuller picture without being overinclusive. When used within a sequential approach, the DAWBA package may help develop a shared language between primary care and specialised care professionals and parents, just as the DAWBA package also produces a report for parents when requested.^46^ This, in turn, might stimulate fruitful discussions within families and help ameliorate discrepancies between the problem perceptions of minors versus caregivers, the perceived focus of treatment and treatment outcomes.^1,44,47,48^ Moreover, a harmonised sequential diagnostic approach might facilitate real integration and joint working in the primary–secondary care interface, a challenge that has not been overcome despite decades of research and dissemination of the importance of EBA. The idea of working within and toward a complete and reliable work-up might be more palatable compared with choosing from a list of measures purely based on one's own familiarity and time limits, without any insight regarding subsequent steps.^6,48^ Earlier studies found the DAWBA to be relatively conservative in terms of the number of diagnoses made and required administration time when compared with other elaborate diagnostic instruments.^25^ This suggests that it might hold potential for use at the primary–secondary care interface, as a second step for those with high scores on screening instruments in primary care and to prioritise referrals and registration in secondary mental healthcare.

In conclusion, our results suggest that integrating referral letters, screening questionnaires and information obtained from assessment is likely to facilitate diagnosis in clinical practice. Prospective studies could further quantify the clinical and economic value of this type of multi-tiered approach, in relation to the facilitation of psychometrically sound and feasible decision-making, timely recognition of problems, determination of required care intensities and treatment outcomes.

## Data Availability

No additional data for this study are available in repositories. Inquiries concerning the data may be made to the corresponding author, S.A.

## Supplementary material

For supplementary material accompanying this paper visit http://doi.org/10.1192/bjo.2022.47. click here to view supplementary material
