# Supplementary material for: The diagnostic process from primary care to child and adolescent mental healthcare services: the incremental value of information conveyed through referral letters, screening questionnaires and structured multi-informant assessment
Source: BJPsych Open. 2022 Apr 7;8(3):e81. doi: 10.1192/bjo.2022.47 (PMC9059622; doi:10.1192/bjo.2022.47)
Supplement: Supplementary file 1 [file S2056472422000473sup001.docx]

# Supplementary material

| Supplementary table 1  Two by two cross-tabulation of the instruments per disorder group presenting positive predictive values | | | | | | | | | | | | | | | | | |
| --- | --- | --- | --- | --- | --- | --- | --- | --- | --- | --- | --- | --- | --- | --- | --- | --- | --- |
|  |  | | | **Anxiety disorders** | | **Depressive disorders** | | **ASD** | | | **ADHD** | | | | **Behaviour disorders** | | |
|  |  | | | **+** | **-** | **+** | **-** | **+** | | **-** | | **+** | **-** | | **+** | | **-** |
| RL | | **+** | 38 (31.9) | | 81 (68.1) | 39 (34.8) | 73 (65.2) | 108 (54.8) | 89 (45.2) | | | 114 (53.5) | 99 (46.5) | 26 (14.3) | | 156 (85.7) | |
|  | | **-** | 43 (8.0) | | 492 (92.0) | 26 (4.8) | 516 (95.2) | 89 (19.8) | 361 (80.2) | | | 90 (20.5) | 350 (79.5) | 18 (3.8) | | 455 (96.2) | |
| SDQ | | **+** | 77 (14.8) | | 442 (85.2) | 62 (11.9) | 457 (88.1) | 140 (40.7) | 204 (59.3) | | | 181 (44.0) | 230 (56.0) | 38 (10.4) | | 328 (89.2) | |
|  | | **-** | 4 (3.0) | | 131 (97.0) | 3 (2.2) | 132 (97.8) | 57 (18.8) | 246 (81.2) | | | 23(9.5) | 219 (90.5) | 6 (2.1) | | 283 (97.9) | |
| Band | | **+** | 57 (23.6) | | 185 (76.4) | 45 (32.4) | 94 (67.6) | 18 (78.3) | 5 (21.7) | | | 121 (60.8) | 78 (39.2) | 16 (6.6) | | 225 (93.4) | |
|  | | **-** | 24 (5.8) | | 388 (94.2) | 20 (30.8) | 495 (96.1) | 179 (28.7) | 445 (71.3) | | | 83 (18.3) | 371 (81.7) | 28 (6.8) | | 384 (93.2) | |
| CR | | **+** | 62 (24.2) | | 194 (75.8) | 49 (32.0) | 104 (68.0) | 151 (49.5) | 154 (50.5) | | | 170 (51.8) | 158 (48.2) | 26 (11.5) | | 200 (88.5) | |
|  | | **-** | 19 (4.8) | | 379 (95.2) | 16 (3.2) | 485 (96.8) | 46 (13.5) | 296 (86.5) | | | 34 (10.5) | 291 (89.5) | 18 (4.2) | | 411 (95.8) | |

Frequency (%) of the positive and negative indications made per instruments and per disorder group, as a ratio of the total number of positive and negative indications made in the considering instrument. Number of diagnoses and sample size were as follows: anxiety disorders n=81 and N=654; depressive disorder n=65 and N=654, autism spectrum disorders (ASD) n=197 and N=647; attention deficit hyperactivity disorder (ADHD) n=204 and N=653; behaviour disorders n=44 and N=655.

Supplementary Figure 1 Diagnostic trajectory

Number (%) of cases detected through RLs, SDQ, DAWBA band, and CR scores, per disorder group, as a ratio of the total frequency of the positive RLs (figure on the left) or negative RLs (right). The continuous lines present inflow, i.e. those that score positive on the concerning instrument. The dotted lines present outflow, i.e. those that score negative on the concerning instrument. Computed in the dataset with complete datapoints dataset N= Anxiety: 654, Depression: 654, ASD:647, ADHD: 551, Behaviour: 655.

Anxiety disorders

Depressive disorders

Autism Spectrum Disorders

ADHD

Behavioural disorders
